# Author Correction: Butterfly-shaped magnetoresistance in triangular-lattice antiferromagnet Ag_2_CrO_2_

**DOI:** 10.1038/s41598-020-61540-y

**Published:** 2020-03-18

**Authors:** Hiroki Taniguchi, Mori Watanabe, Masashi Tokuda, Shota Suzuki, Eria Imada, Takashi Ibe, Tomonori Arakawa, Hiroyuki Yoshida, Hiroaki Ishizuka, Kensuke Kobayashi, Yasuhiro Niimi

**Affiliations:** 1 Department of Physics, Graduate School of Science, Osaka University, Toyonaka, 560-0043 Japan; 2 Center for Spin Research Network, Osaka University, Toyonaka, 560-8531 Japan; 3 Department of Physics, Graduate School of Science, Hokkaido University, Sapporo, 060-0810 Japan; 4 Department of Applied Physics, Graduate School of Engineering, The University of Tokyo, Bunkyo, Tokyo 113-8656 Japan; 5 Department of Physics, Graduate School of Science, The University of Tokyo, Tokyo, 113-0033 Japan; 6 Institute for Physics of Intelligence, Graduate School of Science, The University of Tokyo, Tokyo, 113-0033 Japan

Correction to: *Scientific Reports* 10.1038/s41598-020-59578-z, published online 13 February 2020

In Figure 5, one line of spin down is missing. The correct Figure 5 appears below as Figure 1.

**Figure 1:**
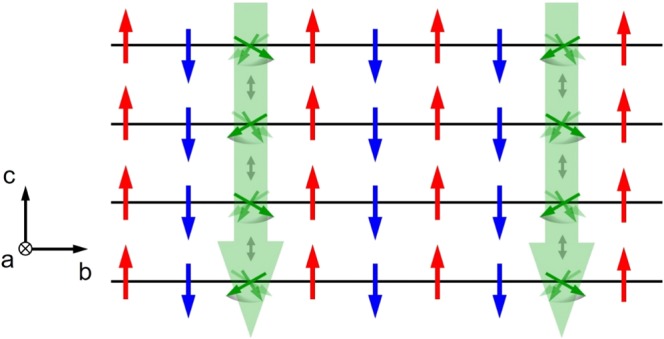
.

