# Supplementary material for: Butterfly-shaped magnetoresistance in triangular-lattice antiferromagnet Ag2CrO2
Source: Sci Rep. 2020 Feb 13;10:2525. doi: 10.1038/s41598-020-59578-z (PMC7018778; doi:10.1038/s41598-020-59578-z)
Supplement: Supplementary file 1 — Supplementary Information. [file 41598_2020_59578_MOESM1_ESM.pdf]

Butterfly-shaped magnetoresistance in triangular-lattice antiferromagnet Ag_2CrO_2

Hiroki Taniguchi,[1] Mori Watanabe,[1] Masashi Tokuda,[1] Shota Suzuki,[1]
Eria Imada,[1] Takashi Ibe,[1] Tomonori Arakawa,[1,2] Hiroyuki Yoshida,[3]
Hiroaki Ishizuka,[4] Kensuke Kobayashi,[1,5,6] and Yasuhiro Niimi[1,2,*]

[1]*Department of Physics, Graduate School of Science, Osaka University, Toyonaka 560-0043, Japan*
[2]*Center for Spintronics Research Network, Osaka University, Toyonaka 560-8531, Japan*
[3]*Department of Physics, Graduate School of Science, Hokkaido University, Sapporo 060-0810, Japan*
[4]*Department of Applied Physics, University of Tokyo, Bunkyo, Tokyo 113-8656, Japan*
[5]*Department of Physics, Graduate School of Science, The University of Tokyo, Tokyo 113-0033, Japan*
[6]*Institute for Physics of Intelligence, Graduate School of Science, The University of Tokyo, Tokyo 113-0033, Japan*

(Dated: January 6, 2020)

*Electronic address: niimi@phys.sci.osaka-u.ac.jp

In this Supplementary information, we show our theoretical model to explain the butterfly-shaped magnetoresistance (MR). As mentioned in the main text, we start with a 2D ferromagnetic spin system with the Ising anisotropy [see Eq. (1)]. Due to this uniaxial anisotropy, a spin gap with the anisotropy energy 2Δ is generated, as shown in Fig. 4. For the positive magnetization, the spin gap increases (decreases) by applying the positive (negative) magnetic field. At $B = B_{\rm c}(< 0)$, the spin gap becomes zero and the spin flip takes place.

We expand the model in Eq. (1) to the quadratic order in S^x and S^y, assuming the S^x and S^y components are sufficiently small. Within this approximation, Eq. (1) reads:

$$\begin{aligned} H &\sim \sum_{\vec{k}} \left[2J\left\{2-\cos(k_x)-\cos(k_y)\right\}+2\Delta+B\right]\left(S^x_{\vec{k}}S^x_{-\vec{k}}+S^y_{\vec{k}}S^y_{-\vec{k}}\right), \\ &\sim \sum_{\vec{k}} \omega_{\vec{k}}\left(S^x_{\vec{k}}S^x_{-\vec{k}}+S^y_{\vec{k}}S^y_{-\vec{k}}\right), \end{aligned} \tag{S1}$$

where $J > 0$ is the ferromagnetic exchange coupling between the nearest neighbor sites, $\Delta > 0$ is the Ising anisotropy, B is the applied magnetic field along the z-direction, $\vec{k}$ is the wave vector, and $\omega_{\vec{k}} = Jk^2 + 2\Delta + B$ is the energy of the spin wave with momentum $\vec{k}$. Using Eq. (S1), we calculate the relaxation time originating from spin fluctuations, i.e., $\tau_{\rm mag}$. The MR due to the spin fluctuations is theoretically expressed with the B dependence of $\tau_{\rm mag}$:

$$\frac{\rho_{xx}(B)-\rho_{xx}(0)}{\rho_{xx}(0)} = \tau(0)\left(\frac{1}{\tau_{\rm mag}(B)}-\frac{1}{\tau_{\rm mag}(0)}\right), \tag{S2}$$

$\tau_{\rm mag}(B)$ is the relaxation time contribution by the magnetic scattering and $\frac{1}{\tau(B)} = \frac{1}{\tau_{\rm mag}(B)} + \frac{1}{\tau_{\rm imp}}$ is the quasi-particle relaxation time ($\tau_{\rm imp}$ is the relaxation time by impurity scatterings). We here assumed that the impurity scattering is insensitive to B; the MR is a consequence of the B dependence of $\tau_{\rm mag}(B)$.

$\tau_{\rm mag}$ is obtained by using the scattering matrix $W_{\vec{k}\alpha\to\vec{k}'\beta}$:

$$\frac{1}{\tau^{\alpha}_{\rm mag}(B)} = \sum_{\beta,\vec{k}'} W_{\vec{k}\alpha\to\vec{k}'\beta}\left(1-\frac{v^x_{\vec{k}'\beta}}{v^x_{\vec{k}\alpha}}\right). \tag{S3}$$

where $v^x_{\vec{k}\alpha} = k_x/m$ is the velocity of electrons with momentum $\vec{k}$ and spin α (m is the mass of an electron and $\hbar = 1$). Here we assume that the electric field is applied along the x-direction and the electron dispersion $\varepsilon_{\vec{k}} = k^2/(2m)$. Within the first Born approximation,

$W_{\vec{k}\alpha\to\vec{k}'\beta}$ reads:

$$W_{\vec{k}\alpha\to\vec{k}'\beta} \sim \frac{2\pi J_{\rm K}^2}{N}\left\langle\left(\delta\vec{S}_{\vec{k}-\vec{k}'}\cdot\vec{\sigma}_{\beta\alpha}\right)\left(\delta\vec{S}_{\vec{k}'-\vec{k}}\cdot\vec{\sigma}_{\alpha\beta}\right)\right\rangle\delta\left(\varepsilon_{\vec{k}\alpha}-\varepsilon_{\vec{k}'\beta}\right),$$
$$\sim \frac{2\pi J_{\rm K}^2}{N}\delta_{\alpha\bar{\beta}}\left\langle S^x_{\vec{k}-\vec{k}'}S^x_{\vec{k}'-\vec{k}}+S^y_{\vec{k}-\vec{k}'}S^y_{\vec{k}'-\vec{k}}\right\rangle\delta\left(\varepsilon_{\vec{k}\alpha}-\varepsilon_{\vec{k}'\beta}\right),$$

where $\delta\vec{S}_{\vec{k}} \equiv \vec{S}_{\vec{k}} - S\hat{z}$, $\vec{S}_{\vec{k}} \equiv \frac{1}{\sqrt{N}}\sum_i \vec{S}_i e^{i\vec{k}\cdot\vec{R}_i}$, and $J_{\rm K}$ is the Kondo coupling between the itinerant electrons and the localized moments. The magnitude of spin fluctuation can be calculated within the linear spin wave approximation:

$$\langle S^{x,y}_{\vec{k}}S^{x,y}_{-\vec{k}}\rangle = \frac{1}{2\beta\omega_{\vec{k}}},$$

where $\beta = 1/T$ is the inverse temperature ($k_{\rm B} = 1$) and a relation $\omega_{\vec{k}} = \omega_{-\vec{k}}$ is assumed. Therefore, $W_{\vec{k}\alpha\to\vec{k}'\beta}$ becomes:

$$W_{\vec{k}\alpha\to\vec{k}'\beta} \sim \frac{2\pi J_{\rm K}^2 S}{N\beta\omega_{\vec{k}'-\vec{k}}}\delta_{\alpha\bar{\beta}}\delta\left(\varepsilon_{\vec{k}\alpha}-\varepsilon_{\vec{k}'\beta}\right). \tag{S4}$$

By substituting Eq. (S4) into Eq. (S3), the relaxation time due to spin fluctuation can be obtained as follows:

$$\frac{1}{\tau^\alpha_{\rm mag}(B)} = 2\pi J_{\rm K}^2 ST\int\frac{dk'^2}{(2\pi)^2}\frac{1}{\omega_{\vec{k}'-\vec{k}}}\left(1-\frac{v^x_{\vec{k}'\bar{\alpha}}}{v^x_{\vec{k}\alpha}}\right)\delta(\varepsilon_{\vec{k}\alpha}-\varepsilon_{\vec{k}'\bar{\alpha}}). \tag{S5}$$

By integrating over the Fermi surface, Eq. (S5) reads:

$$\frac{1}{\tau^\alpha_{\rm mag}(B)} = \frac{J_{\rm K}^2 SmT}{2\pi}\int\frac{d\theta}{J\{(k_{\rm F}^\alpha)^2+(k_{\rm F}^{\bar{\alpha}})^2-2k_{\rm F}^\alpha k_{\rm F}^{\bar{\alpha}}\cos\theta\}+2\Delta+B}\left(1-\frac{k_{\rm F}^{\bar{\alpha}}}{k_{\rm F}^\alpha}\cos\theta\right),$$
$$= \frac{J_{\rm K}^2 SmT}{2Jk_{\rm F}^\alpha k_{\rm F}^{\bar{\alpha}}}\left[F_1\left(\frac{k_{\rm F}^\alpha}{2k_{\rm F}^{\bar{\alpha}}}+\frac{k_{\rm F}^{\bar{\alpha}}}{2k_{\rm F}^\alpha}+\frac{2\Delta+B}{2Jk_{\rm F}^\alpha k_{\rm F}^{\bar{\alpha}}}\right)-\frac{k_{\rm F}^{\bar{\alpha}}}{k_{\rm F}^\alpha}F_2\left(\frac{k_{\rm F}^\alpha}{2k_{\rm F}^{\bar{\alpha}}}+\frac{k_{\rm F}^{\bar{\alpha}}}{2k_{\rm F}^\alpha}+\frac{2\Delta+B}{2Jk_{\rm F}^\alpha k_{\rm F}^{\bar{\alpha}}}\right)\right]. \tag{S6}$$

Here the two functions F_1 and F_2 are $F_1(x) = \dfrac{1}{\sqrt{x^2-1}}$ and $F_2(x) = \dfrac{x}{\sqrt{x^2-1}} - 1$, respectively; $\alpha(=\uparrow$ or $\downarrow)$ and $\bar{\alpha}(=\downarrow$ or $\uparrow)$ denote the spin directions, and $k_{\rm F}^\alpha$ is the Fermi wave number for spin α. Equation (S6) shows that the relaxation time due to spin fluctuation depends only on the Fermi wave number.

Since Ag_2CrO_2 has a small magnetic moment, it is reasonable to assume $k_{\rm F}^\uparrow \sim k_{\rm F}^\downarrow \sim k_{\rm F}$. Therefore, we obtain the following expression for $\tau_{\rm mag}$:

$$\frac{\hbar}{\tau_{\rm mag}(B)} \sim \frac{J_{\rm K}^2 Smk_{\rm B}T}{2Jk_{\rm F}^2\hbar^2}\left\{F_1\left(1+\frac{2\Delta+\mu_{\rm eff}B}{2Jk_{\rm F}^2a_0^2}\right)-F_2\left(1+\frac{2\Delta+\mu_{\rm eff}B}{2Jk_{\rm F}^2a_0^2}\right)\right\}, \tag{S7}$$

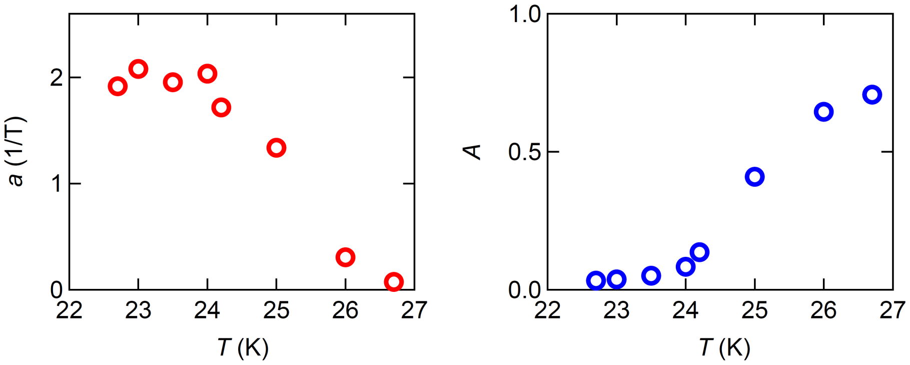

Figure S1: (a) A and (b) a obtained from the fitting with Eq. (S8).

which is the same as Eq. (2) in the main text. k_F is the Fermi wave number, μ_{eff} is the effective ferromagnetic moment, a_0 is the lattice constant between the neighboring effective ferromagnetic moments. In Eq. (S7), $\hbar$ and k_{B} are explicitly written here to compare the experimental data. To fit the experimentally obtained MR curves with Eqs. (S2) and (S7), the following expression is useful:

$$\frac{\rho_{xx}(B)-\rho_{xx}(0)}{\rho_{xx}(0)} = A\left\{\sqrt{\frac{aB_{\mathrm{c}}}{aB_{\mathrm{c}}+2}}-\sqrt{\frac{a(B+B_{\mathrm{c}})}{a(B+B_{\mathrm{c}})+2}}\right\}, \tag{S8}$$

where A and a are fitting parameters. As shown in Fig. 2(e), the experimental data are fitted using Eq. (S8). We note that in principle Eq. (S8) is valid only in the vicinity of T_{N}. Figure S1 shows the obtained A and a values as a function of T. A is small below T_{N} and rapidly increases near T_{N}. On the other hand, a is more or less constant below T_{N} and rapidly decreases above T_{N}.

Now we roughly estimate J and Δ by using the following relations:

$$J = \frac{\mu_{\mathrm{eff}}}{2ak_{\mathrm{F}}^2a_0^2}, \tag{S9}$$

$$\Delta = \frac{B_{\mathrm{c}}\mu_{\mathrm{eff}}}{2}. \tag{S10}$$

It should be stressed that Eqs. (S9) and (S10) give rough estimations for J and Δ, since in principle J and Δ should depend on all the microscopic details. As mentioned in the main text, the uniform magnetic moment is along the c-axis. In addition, we assume that the exchange interaction along the c-axis is much larger than that in the plane. Such a situation is common for antiferromagnets with partially disordered phases [31-33] and is necessary for

a stable PD phase in Monte Carlo simulations [34, 35]. The strong exchange interaction along the c-axis develops strong correlation of spins along the c-axis. Therefore, we assume that the quasi-one-dimensional chain along the c-axis behaves as a large single spin in our 100 nm thick sample. Since the lattice constant of the c-axis is 0.866 nm, the total effective magnetic moment μ_{eff} along the c-axis is estimated to be $\frac{100}{0.866} \times 0.4\mu_{\mathrm{B}} \sim 45\mu_{\mathrm{B}}$ where $0.4\mu_{\mathrm{B}}$ is the effective magnetic moment per unit cell [21]. By assuming $k_{\mathrm{F}}a_0 \sim 1$ (as in the case of a typical metal), both J and Δ are estimated to be about 10 K, which is reasonable for the present case.
